# A Morphometric Study of the Foramen of Diaphragma Sellae and Delineation of Its Relation to Optic Neural Pathways through Computer Aided Superimposition

**DOI:** 10.1155/2015/618042

**Published:** 2015-09-13

**Authors:** Doris George Yohannan, Renuka Krishnapillai, Romi Suresh, Shobha Ramnarayan

**Affiliations:** ^1^Department of Anatomy, Sree Gokulam Medical College and Research Foundation, Venjaramoodu, Thiruvananthapuram, Kerala 695607, India; ^2^Department of Anatomy, Government TD Medical College, Alappuzha, Kerala 688005, India; ^3^Department of Anatomy, Government Medical College, Thiruvananthapuram, Kerala 695011, India

## Abstract

The diaphragma sellae (DS) is a fold of dura that forms a partial roof over the pituitary gland. The foramen of the diaphragma sellae (FDS) is thereby a pathway for suprasellar extension of pituitary tumors. The purpose of this study was to describe the anatomical dimensions of the DS and FDS and to understand the relationship of FDS with the overlying optic chiasma. The study was conducted in 100 autopsy cases. Measurements were taken using vernier calipers. Photographs, taken before and after removal of optic pathway, were superimposed using image processing software. The results showed that the mean A-P dimension of DS was 1.17 ± 0.48 cm; the lateral dimension of DS was 1.58 ± 0.60 cm. The mean A-P dimension of FDS was 0.66 ± 0.42 cm; the lateral dimension of FDS was 0.82 cm ± 0.54 cm. The shapes of FDS were irregular (40%), transversely oval (29%), circular (13%), sagittally oval (11%), or trapezoid with posterior dimension more than the anterior one (6%) or anterior dimension more than the posterior one (1%). The margins of FDS were either well defined (31%) or ill defined (69%). The positional relation of FDS to optic chiasma was also found out.

## 1. Introduction

The diaphragma sellae (DS), the fold of dura that lies over the sphenoid bone forming a partial roof over the pituitary gland, is an important landmark in the transcranial and endoscopic approaches of pituitary surgeries. The role of the dura, being a thickened barrier and thereby potentially directing the growth of pituitary tumors through its weaknesses and deficiencies, has also been proven in previous studies [1]. Hence the role of the position and the morphometry of the foramen of diaphragma sellae (FDS) become important in directing the growth of a mass lesion in the sellar region. The position of the optic chiasma with relation to the FDS hence becomes important, playing a role in the clinical presentations of pituitary tumors and also as a structure which has great importance in the surgery of pituitary. Though many textbooks mention the FDS and its relationship to the optic chiasma, they have shown considerable variations in many studies [2–5]. This study is conducted to clarify further the anatomical aspects of the suprasellar area. The clinical implication of the study is that it will help in defining the structure of DS and FDS, thus leading us to predict, more precisely, the effects of extension of pituitary adenomas, especially the effects on compressing different parts of the visual pathway.

## 2. Materials and Methods

The study was conducted on 100 adult autopsy specimens obtained from the Department of Forensic Medicine, Government Medical College, Thiruvananthapuram. Cases with history or examination findings of intracranial injuries or intracranial pathologies and cases with signs of tissue decomposition were excluded from the sample. Institutional research technical committee and ethical committee clearance were obtained after submitting the protocol, prior to the study.

The observations were conducted at the time of autopsy. Consent was taken from the relatives of the deceased.

Certain precautions were taken at the time of autopsy. Care was taken not to injure or cut the optic nerves or optic chiasma and an incision was made as posteriorly as possible in the optic tract before it winds the midbrain in the perimesencephalic cistern. So when the brain was removed, the visual pathway remained on the floor of the cranial fossa over the diaphragma sellae. This gave adequate exposure to visualize both the optic neural pathways and the diaphragma sellae.

A photograph was taken from the cranial aspect using a standard digital camera with adequate natural lighting (flash use was disabled). After these, the optic nerves were incised anteriorly and removed along with the optic chiasma. This brings the underlying DS and the FDS into view.

**Figure 1:**
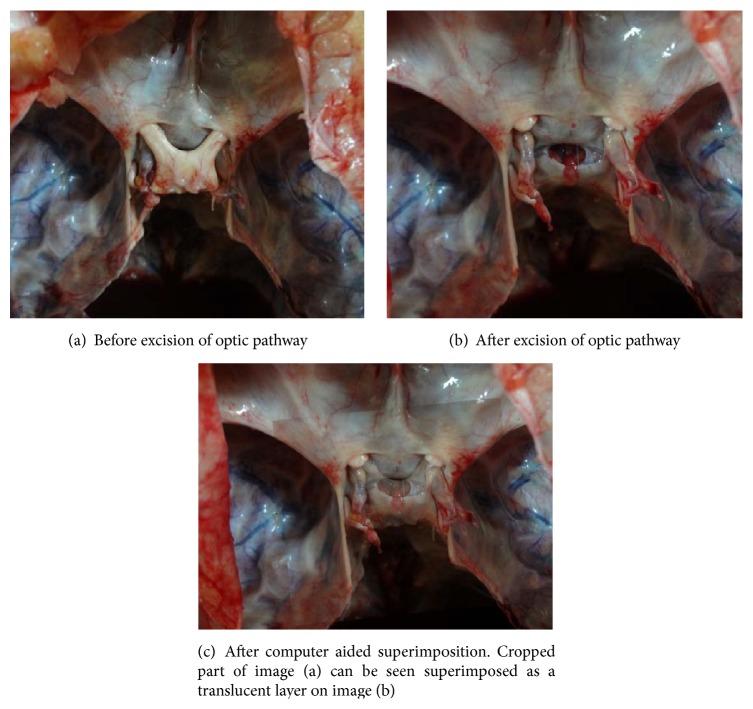
Computer screen capture image showing superimposition, in image processing software, of the two images of the cranial fossa obtained (a) before and (b) after removing the optic chiasma and (c) the superimposed image.

The measurements of the DS (anteroposterior and lateral) and the FDS (anteroposterior and lateral) were measured using vernier calipers (least count, 0.1 mm). The shape of the FDS was noted. Again a photograph was taken in the same manner as the first one, but this one excludes the visual pathways, giving us the view of the DS and FDS. As both of these photographs were taken in the same manner, the two photographs, each of the 100 samples taken, were sorted and copied onto a computer hard disk and loaded onto an image processing software and the two images were superimposed as two layers (see Figure 1). The technique of software aided superimposition allows us to comment exactly, without subjective variability, about the position of the FDS with respect to optic chiasma.

## 3. Results

### 3.1. Diaphragma Sellae

#### 3.1.1. Anteroposterior Dimension of DS

The anteroposterior dimension of the DS was analysed. The mean value was found to be 1.17 cm with a standard deviation of 0.24 cm. The lowest value observed was 0.80 cm and the highest value was 1.83 cm (see Table 1).

#### 3.1.2. Lateral Dimension of DS

The lateral dimension of the DS was analysed. The mean value was found to be 1.58 cm with a standard deviation of 0.30 cm. The lowest value observed was 0.74 cm and the highest value was 2.46 cm (see Table 2).

### 3.2. Foramen of the Diaphragma Sellae

#### 3.2.1. Anteroposterior Dimension of FDS

The anteroposterior dimension of the FDS was analysed. The mean value was found to be 0.66 cm with a standard deviation of 0.21 cm. The lowest value observed was 0.23 cm and the highest value was 1.40 cm (see Table 3).

#### 3.2.2. Lateral Dimension of FDS

The lateral dimension of the FDS was analysed. The mean value was found to be 0.82 cm with a standard deviation of 0.27 cm. The lowest value observed was 0.25 cm and the highest value was 1.52 cm (see Table 4).

### 3.3. Shape of the FDS

**Figure 2:**
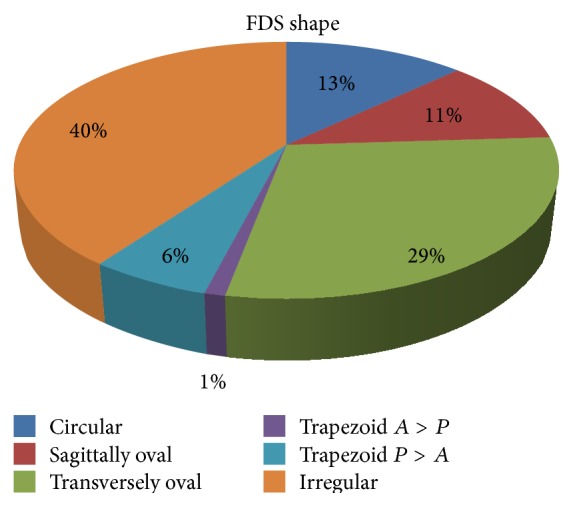
Figure showing proportion of various shapes of FDS.

The shapes of the FDS were observed and analysed. Their frequency distribution was demonstrated (Figure 2).

**Figure 3:**
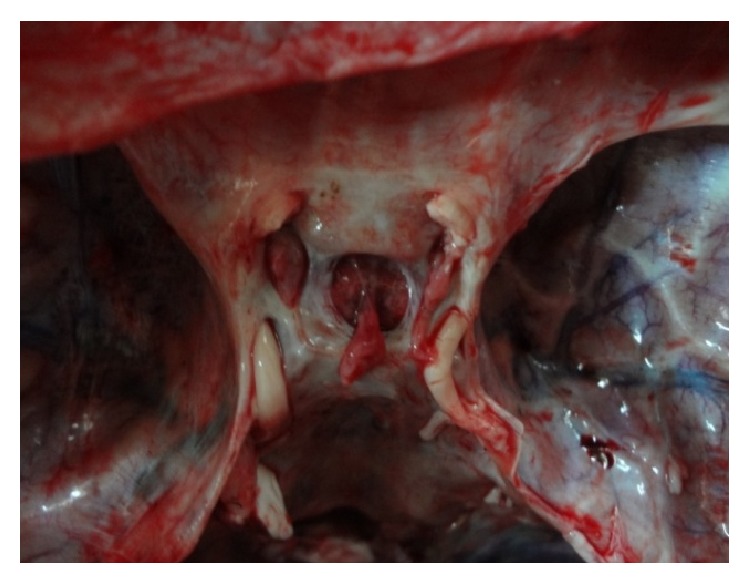
Photograph showing a* circular* shaped FDS.

**Figure 4 fig4:**
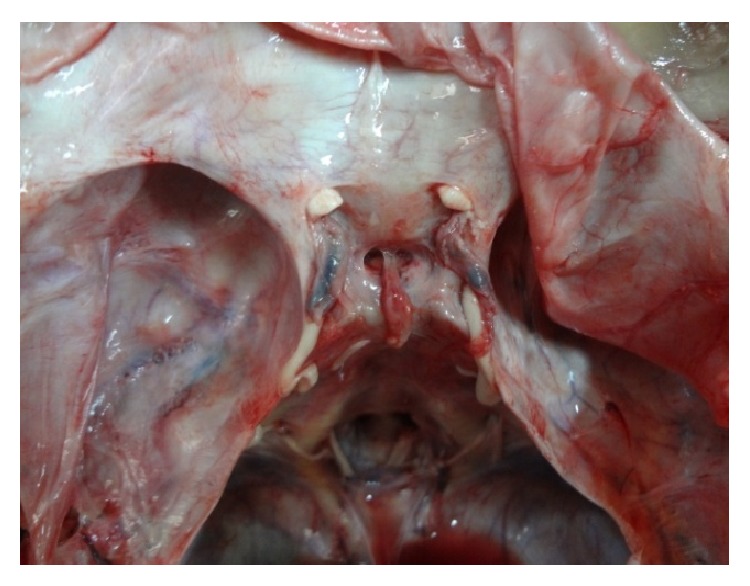
Photograph showing a* transversely oval* shaped FDS.

**Figure 5 fig5:**
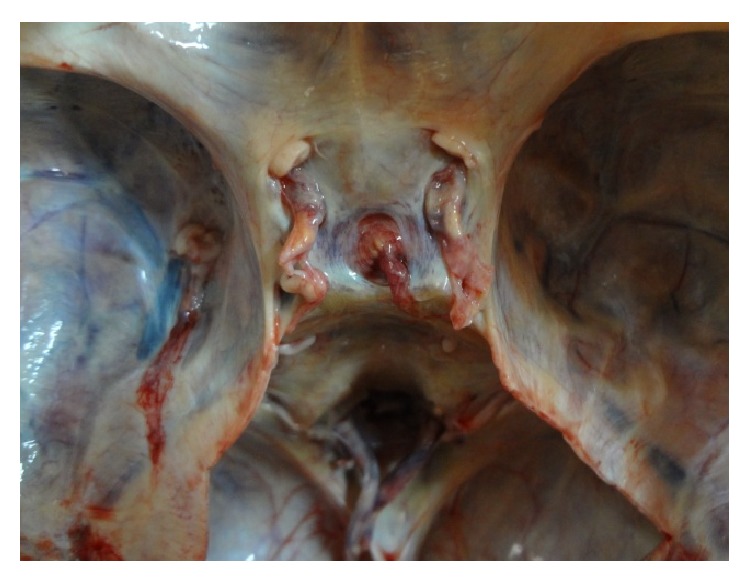
Photograph showing a* sagittally oval* shaped FDS.

**Figure 6 fig6:**
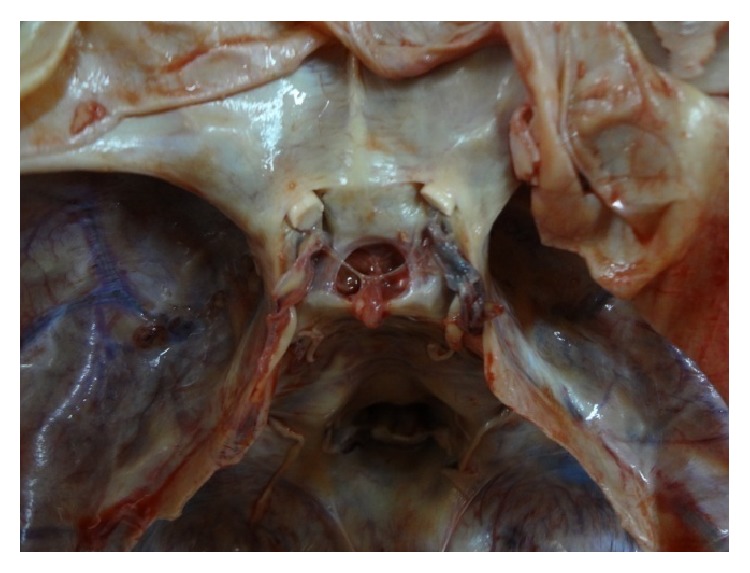
Photograph showing a* trapezoid* (*posterior* >* anterior*) shaped FDS.

**Figure 7 fig7:**
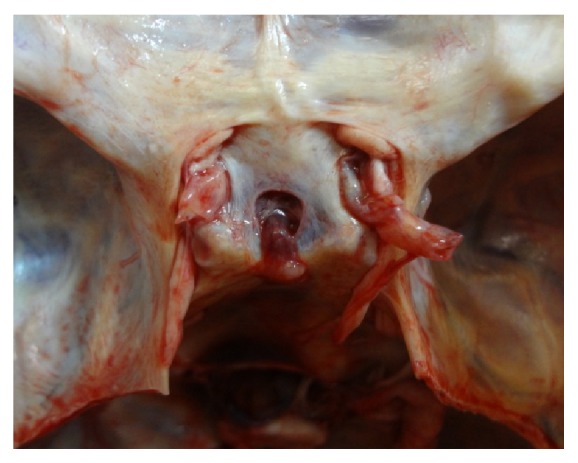
Photograph showing a* trapezoid* (*anterior* >* posterior*) shaped FDS.

**Figure 8:**
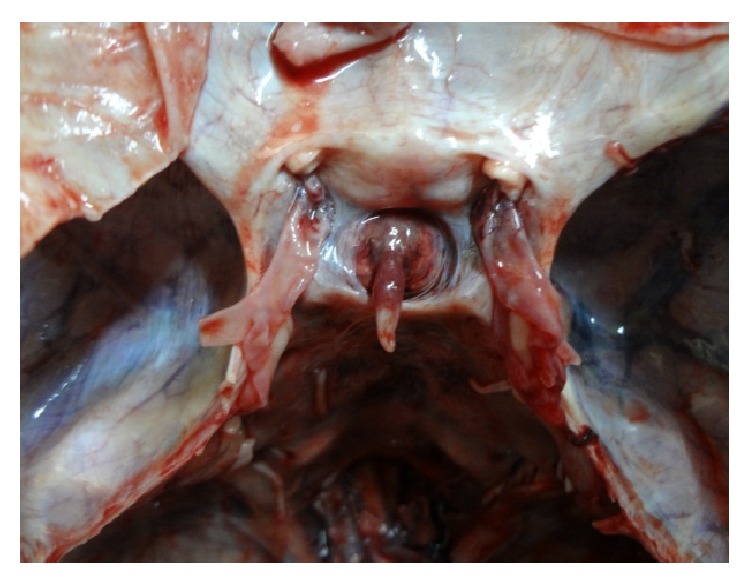
Photograph showing an* irregularly* shaped FDS.

The main shapes are demonstrated in original photographs (see Figures 3 [Fig fig4] [Fig fig5] [Fig fig6] [Fig fig7]–8).

### 3.4. Margin of FDS

**Figure 9:**
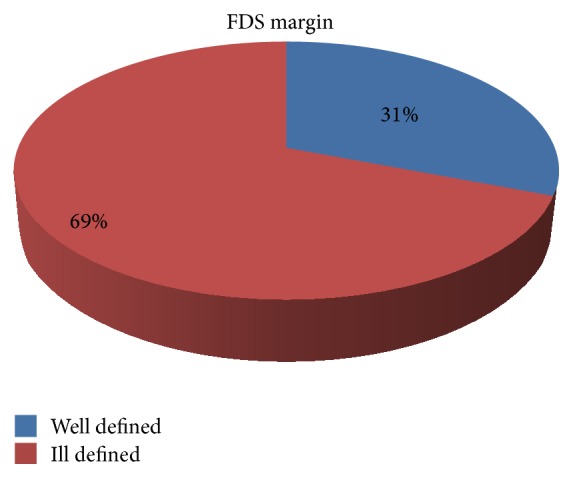
Figure showing proportions of well and ill defined margins of FDS.

**Figure 10:**
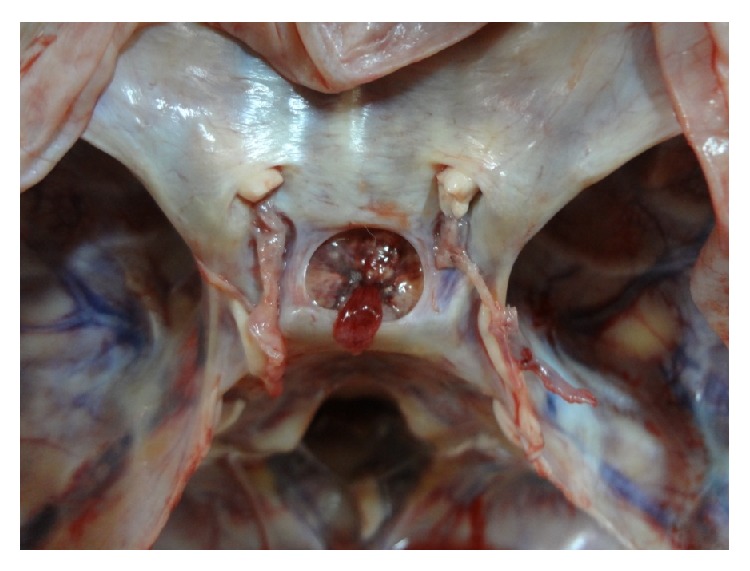
Photograph showing a well defined margin of FDS.

**Figure 11:**
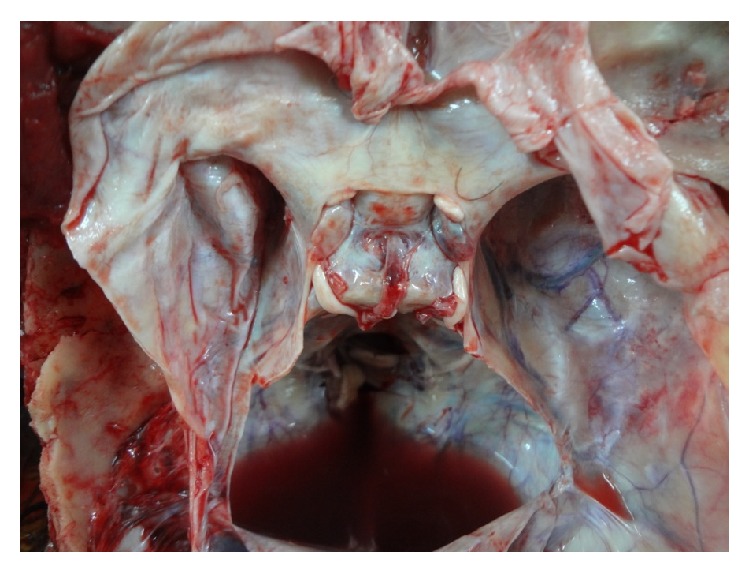
Photograph showing an ill defined margin of FDS.

The margins of the FDS were described as well defined or ill defined, and their proportions in the study sample were found out (see Figure 9). Specimen photographs are shown in Figures 10 and 11.

### 3.5. Relationship of Optic Chiasma to FDS

**Figure 12:**
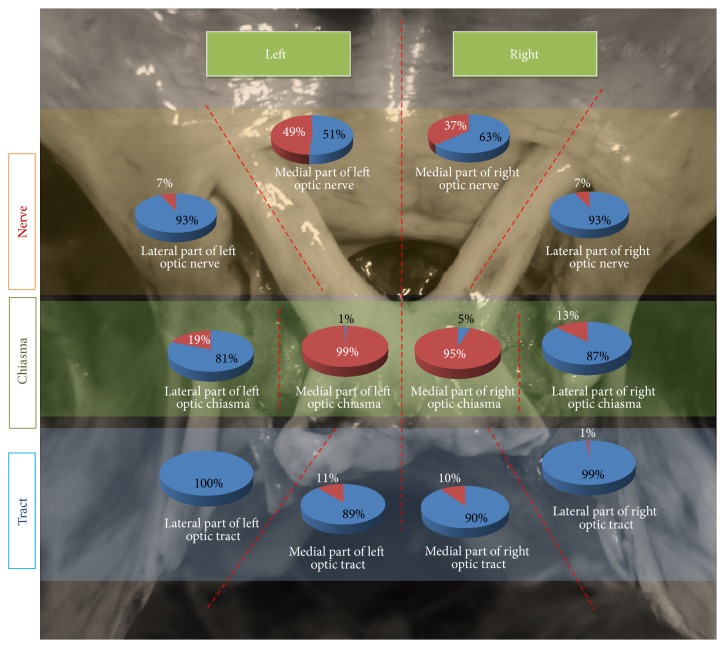
Illustrated diagram showing relationship of parts of optic neural pathways with FDS in the study sample.The red portion in each pie chart represents the percentage of cases where FDS is related to that particular part of optic pathway. For example, in the* top left pie diagram*, in 7% of cases, the* lateral part of left optic nerve* was inferiorly related to FDS.

The position of the optic chiasma was noted with respect to the position of the FDS, with the help of computer aided superimposition. The portions of the optic pathways that were related superiorly to the FDS were noted and analysed. There were 12 categories, that is, related to medial or lateral parts of right or left, optic nerve, chiasma, or tracts. The results are representatively depicted in Figure 12. The optic chiasma was found to completely cover the FDS in 45% of cases. It did not cover the FDS completely in 55%.

## 4. Discussion

### 4.1. Diaphragma Sellae

In the present study the mean anteroposterior measurement of DS was 1.17 ± 0.48 cm (11.74 ± 4.8 mm) with values that ranged from 0.80 to 1.83 cm. In the study by Kursat et al. [6], the sagittal measurements were found to be varied between 6.44 and 11.74 mm. The mean length was 9.55 ± 1.38 mm. The coronal (lateral) plane length of the diaphragma was in the range of 10.62–16.06 mm, with a mean of 13.65 ± 1.66 mm in the study by Kursat et al. In the present study the mean was 1.58 ± 0.58 cm (15.77 ± 5.8 mm) with a range of 0.74 to 2.46 cm.

From the above values we understand that both the lateral and A-P dimensions were more than the ones previously observed by Kursat et al. It suggests that the diaphragma sellae is a dural fold where, in most cases, coronal length was greater than sagittal length. This suggests that the diaphragma sellae is most often a coronally oriented rectangle/ovoid, rather than a circular fold as described in earlier descriptions (see Table 5). The difference in the values observed in the present study is most likely to be due to the better sample size, as opposed to Kursat et al.'s study, which was done in 16 sphenoid block samples.

### 4.2. Foramen of Diaphragma Sellae

#### 4.2.1. Measurements

In the present study the mean A-P measurement of FDS was 0.65 ± 0.42 cm. The values ranged from 0.23, a tight FDS, to 1.40 cm, a very wide FDS. The mean lateral (coronal) FDS measurement was 0.82 ± 0.54 cm. The lowest value was 0.25 cm and the largest value was 1.52 cm. One finding that was observed in these specimens was that as the FDS size increases, the pituitary tissue seen in the sellar space was relatively less. This observation was consistent with the findings in the studies by Ferreri et al. [8] and Kaufman [9], which stresses a possible relationship between the size of the FDS and the pituitary tissue, proposing a developmental and morphological aspect to the pathogenesis of empty sella syndrome. In their landmark study, Bergland et al. [2] describe primary empty sella syndrome as a variant anatomy, rather than a disease. Their study states that FDS with a dimension more than 5 mm will cause primary empty sella syndrome. In their case series, ~40% of cases had defects in DS of 5 mm or more. In >20% of cases there was intrasellar extension of subarachnoid space. Five percent of cases had fully developed empty sella. Variations in dynamics of CSF flow, in the sellar region, in the context of an incompetent diaphragma have also been proposed as a mechanism of empty sella syndrome [10].

The present study measurements were almost consistent with the measurements described in previous studies. The mean width and length of the FDS were 7.9 ± 2.0 and 7.6 ± 1.9 mm, respectively, in the study by Won et al. [5]. They said that both were larger in males than in females (*p* < 0.05), though there was no statistically significant difference of the FDS size based on sex in the present study. The mean width and length of the FDS were 7.33 and 7.26 mm in the study conducted on 20 cadaver heads, by Campero et al. [1]. The mean width and length were 11 and 8 mm in the study by Renn and Rhoton Jr. [7]. In the study by Kursat et al. [6] sagittal (A-P) diameter of the FDS ranged between 3.85 and 8.74 mm with a mean of 6.40 ± 1.30 mm. In the coronal plane, the central opening ranged between 3.68 and 10.42 mm, with a mean coronal diameter of 6.20 ± 2.14 mm (see Table 6).

### 4.3. Shape

In the present study most of the foramina were irregular in shape (40%). The circular variety was seen only in 13%. They were oval with a sagittal orientation in 11% and transverse orientation in 29%. The trapezoid types were seen in 7%. In the study by Won et al. [5], the shape of FDS was noted to be round in 70% or oval in 30%. The shape of the FDS was reported to be more often rectangular than circular by Renn and Rhoton Jr. [7]. In the study by Kursat et al. [6], the FDS had an oval shape in ten (62%), triangular shape in three (19%), and rectangular shape in three (19%) specimens. Such variety of shapes of the FDS found in the present study is not described in previous literature. The findings are useful in neurosurgery as well as in neuroimaging. The different shapes of the foramina give us a clue as to the possibility of diverse patterns of growth of tumors from within the sellar space.

### 4.4. FDS Relation with Optic Neural Pathways

In the study by Won et al. [5] the location of the FDS was classified into the following types: type I (on the midline, 69%), type II (in the anterior and left parts, 22%), and type III (in the anterior and right parts, 9%). Type I was further subdivided into three types: type I-a (in the anterior part, 14%), type I-b (in the anterior, right, and left parts, 33%), and type I-c (in all four parts, 22%)

In the present study we divided it in a more stringent classification which can provide much better information for the surgeon (see Figure 12). The above information gives us pinpoint knowledge as to the possible types of lesions of the visual pathway, which can occur, if the tumor invades the suprasellar area through the deficiency in the diaphragma. The fact that lateral parts of the optic nerve are related to the FDS in 7% on the right and 7% on the left provides chances for tumors to compress the optic nerve completely including the fibres from temporal retina (nasal field). The relation on the medial aspect of optic nerve again creates a chance to compress optic nerve as well as cause a junctional scotoma in the contralateral eye due to the disposition of von Willebrand's knee [11, 12]. The 19% relation of FDS on lateral part of left half of optic chiasma and 13% relation on lateral part of right half of optic chiasma again can potentially direct tumor masses to compress fibres from temporal retina (nasal fields). The medial half of chiasma was related to FDS in 99% of cases in the left and 95% of cases in the right, which is in concurrence with the classic teaching of bitemporal hemianopia as the most common presentation of pituitary tumors with suprasellar extension. The FDS was almost never related to the optic tract completely, which is again in concurrence with the classic teaching that hemianopia is not classically seen in pituitary tumors.

### 4.5. FDS Covered by Chiasma

Optic chiasma completely covered the FDS, in 45% of cases in the present study. In the remaining 55% of cases the FDS could be seen peeping through the angle between the nerves or more laterally covering the optic nerves. In their paper, Won et al. [5] describe that, in around one-third of their cases, that is, 30%, they found the optic chiasma to completely cover the FDS. In a previous study by Hollenhorst and Younge [13] they found it to be 15%. Gray's Anatomy textbook [14] describes that the dorsal surface of the diaphragma sellae is partially covered by the optic chiasma, giving no details about it covering the FDS or not.

## 5. Conclusion

This study, which was done in 100 autopsy samples, provides detailed information about the morphometry of diaphragma sella and its foramen, the gross anatomical features of the foramen, and its topographical relationship to the different parts of optic pathway. The information can help us understand the effect of many disease processes in the sellar region, namely, pituitary tumors, craniopharyngiomas, empty sella syndrome, and arachnoid cysts. The data provides the neurosurgeon with information essential to understand the suprasellar area in more detail. The information is also vital for the neuroradiologist, who needs to assess the state of the chiasma and visual pathways prior to and after any neurological procedure in this key area of the brain.

## Figures and Tables

**Table 1:** Univariate analysis of anteroposterior dimension of diaphragma sellae.

Anteroposterior dimension of diaphragma sellae (cm.)	
Mean	1.17
Std. deviation	0.24
Lowest value observed	0.80
Highest value observed	1.83

**Table 2:** Univariate analysis of lateral dimension of diaphragma sellae.

Lateral dimension of diaphragma sellae (cm.)	
Mean	1.58
Std. deviation	0.30
Lowest value observed	0.74
Highest value observed	2.46

**Table 3:** Univariate analysis of anteroposterior dimension of FDS.

Anteroposterior dimensions of FDS (cm.)	
Mean	0.66
Std. deviation	0.21
Lowest value observed	0.23
Highest value observed	1.40

**Table 4:** Univariate analysis of lateral dimension of FDS.

Lateral dimension of FDS (cm.)	
Mean	0.82
Std. deviation	0.27
Lowest value observed	0.25
Highest value observed	1.52

**Table 5:** Comparison of the studies of dimensions of diaphragma sellae.

Study	A-P dimension		Coronal dimension	
	Mean (mm.)	SD (mm.)	Mean (mm.)	SD (mm.)
Kursat et al. [6]	9.55	0.69	13.65	0.83
**Present study**	11.74	2.4	15.77	2.9

**Table 6:** Comparison of the studies of dimensions of FDS.

Study	A-P dimension		Coronal dimension	
	Mean (mm.)	SD (mm.)	Mean (mm.)	SD (mm.)
Kursat et al. [6]	6.40	0.65	6.20	1.07
Won et al. [5]	7.6	0.95	7.9	1.0
Campero et al. [1]	7.26	—	7.33	—
Renn and Rhoton Jr. [7]	8	—	11	—
**Present study**	6.5	2.1	8.2	2.7
